# Association between Serum Homocysteine Level and Obstructive Sleep Apnea: A Meta-Analysis

**DOI:** 10.1155/2017/7234528

**Published:** 2017-07-31

**Authors:** Kun Li, Jing Zhang, Yanwen Qin, Yong-Xiang Wei

**Affiliations:** Department of Otolaryngology, Beijing Anzhen Hospital, Capital Medical University, Beijing 100029, China

## Abstract

**Background:**

Obstructive sleep apnea (OSA) is a common problem that affects human health. Researches have reported a variety of results with reference to the association between OSA and serum homocysteine (Hcy) level. This meta-analysis is proposed to figure out the association between serum Hcy level and OSA.

**Methods:**

Eligible studies were identified via searching PubMed, Embase, and China National Knowledge Infrastructure (CNKI). Two independent reviewers reviewed studies. The Newcastle-Ottawa Quality Assessment Scale (NOS) was employed for quality assessment of included studies. RevMan (5.1) software and STATA (12.0) software were applied to data analyses.

**Results:**

10 studies containing 839 subjects were included in the present meta-analysis; results revealed that Hcy levels in OSA group were 2.40 *μ*mol/l higher than that in control group (95% confidence interval: 0.6 to 4.20, *P* < 0.01; *I*^2^ = 96%). Subgroup analysis showed a significant increase of serum Hcy level in OSA patients compared with healthy controls when apnea hyperpnoea index (AHI) >= 30.

**Conclusions:**

Serum Hcy levels and OSA have close-knit and significant association. Analyses demonstrated that patients with OSA had a higher serum Hcy level than healthy controls. In addition, this difference is more significant in moderate or severe OSA patients.

## 1. Introduction

Obstructive sleep apnea (OSA) is a common problem affecting 2–4% of the world's population. The main characteristic of OSA is recurrent hypopnea or apnea during sleep, a phenomenon that leads to intermittent hypoxia or frequent episodes of arousal [1]. The time of intermittent hypoxia or arousal may be very long, which may cause severe hypoxemia. These changes might also lead to many medical disorders such as hypotension or coronary heart disease [2, 3]. Therefore, OSA might cause severe complications and brought serious influences to OSA patients' health.

Homocysteine (Hcy) is a sulfur-containing amino acid cysteine synthesized in the liver, and it can participate in the metabolism of methionine [4]. Recently, Hcy has attracted wide attention among researchers, and these researchers have verified that homocysteine had a close relationship with many diseases [5, 6]. The level of blood Hcy is independently linked with a proportional risk of mortality [7]. For instance, Chambers et al. demonstrated that hyperhomocysteinemia weakens endothelial dysfunction and promotes the metabolism of toxic cysteine adducts and maintains oxidative stress [8].

However, the previous studies have shown that the serum homocysteine levels in OSA patients compared with health subjects have not reached a consensus yet [9–18]. Our study sought to demonstrate whether Hcy levels were elevated in OSA patients by pooling all conflicting findings.

## 2. Materials and Methods

### 2.1. Ethics Statement

Our analyses were all based on previously published studies. Hence, our review did not need the ethical approval.

### 2.2. Search Strategy

We searched for studies in PubMed, Embase, and China National Knowledge Infrastructure (CNKI) database from their inception to August 1, 2016, without restrictions. The following search keywords were used: homocysteine, obstructive sleep apnea (OSA), obstructive sleep apnea-hypopnea syndrome (OSAHS), sleep apnea, obstructive sleep apnea syndrome (OSAS), obstructive sleep hypopnea, and sleep-disordered breathing.

### 2.3. Eligibility Criteria

Two independent reviewers identified the selected studies. The inclusion criteria are as follows: all participants were examined by polysomnography (PSG); participants were divided into two groups according to their apnea-hypopnea index (AHI) values (OSA group, AHI >= 5; control group, AHI < 5). The subjects did not receive any treatments, such as CPAP treatment, operations, and so on. No complication was reported for all participants, namely, coronary heart disease, stroke, metabolism syndrome, acute coronary syndrome, and cerebral infarction. The included articles consist of both case-control and cross-sectional studies.

### 2.4. Quality Scale

The quality assessment of included studies was performed using the Newcastle-Ottawa Quality Assessment Scale (NOS). Scores ranged from 5 to 7; relevant details were shown in Table 1.

### 2.5. Data Extraction

Two independent investigators extracted data from the included studies: first author, year of publication, sample size, study population, mean age, and serum Hcy levels in both groups, and another reviewer checked the extracted data for completeness and accuracy.

### 2.6. Statistical Methods

The difference in serum homocysteine levels between the OSA group and the control group was assessed with weighted mean difference (WMD). Moreover, we also stratified the data into three subgroups according to BMI (>=30 and <30) and AHI (>=30 and <30), respectively. Then subgroup data were analyzed separately. *I*-square (*I*^2^) test quantified the heterogeneity of WMDs. When heterogeneity was low (*I*^2^ < 40), fixed-effects model was adopted; otherwise, random-effects model was used. Begg's and Egger's tests were performed for assessing the publication bias [19, 20]. The stability of the meta-analysis was assessed using a sensitivity analysis. We performed metaregression to confirm the probable sources of heterogeneity. RevMan (5.1) software and STATA (12.0) software were used for data analysis, and *P* < 0.05 was defined as being statistically significant. The statistical power of our study was measured by PASS 11.0.

## 3. Results

### 3.1. Search Results

**Figure 1:**
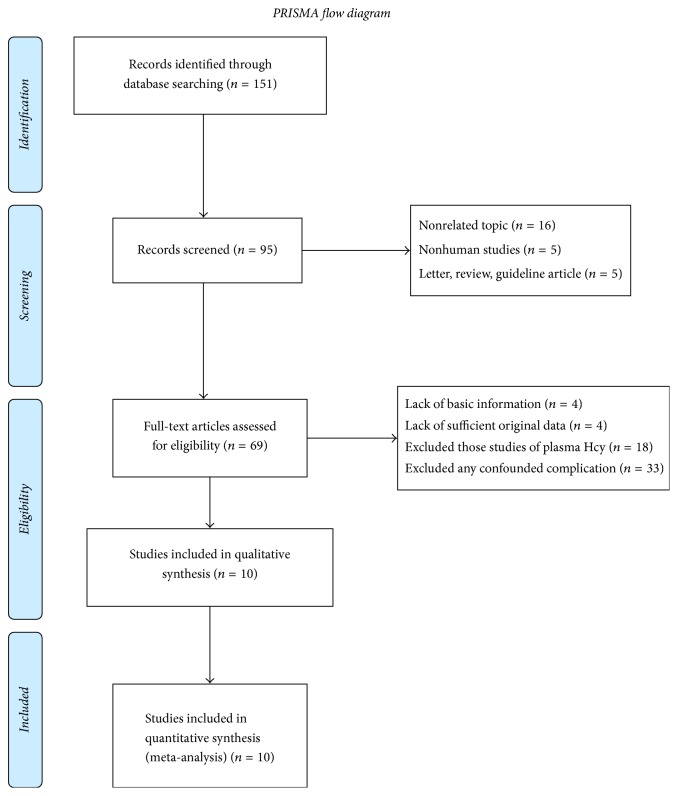
Flow diagram of the study search. Flow chart of the study selection process. After careful discussion between the 2 reviewers, a total of 10 studies were included to perform the meta-analysis.

We initially obtained 105 studies from PubMed, Embase, and CNKI. After the review of titles and abstracts, 36 studies were excluded. Then another 59 articles are excluded for reasons shown in Figure 1. Finally, our meta-analysis included 10 studies.

### 3.2. Characteristics of the Eligible Studies

Our meta-analysis included 10 studies containing 773 participants [9–18]. 457 out of the 773 participants are OSA patients. According to the current sample size and other information, our study has sufficient statistical power (Power > 0.95). Table 1 contained first author's name, year of publication, sample size, country, and the value of NOS.  Table 2 showed information about mean age, AHI, BMI, and serum Hcy levels.

### 3.3. Pooled Analysis

**Figure 2:**
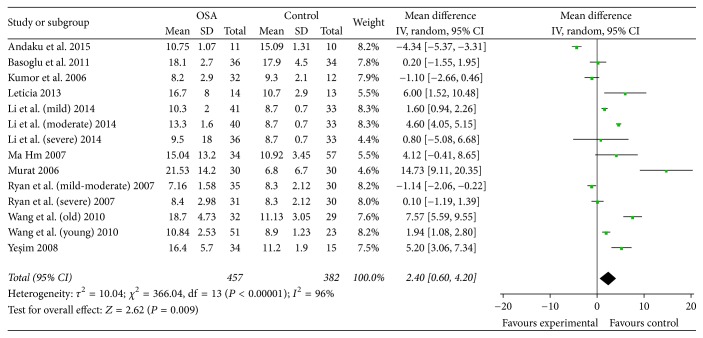
Meta-analysis and forest plot. Calculation based on random-effects model. Results are expressed as weighted mean difference (WMD) and 95% confidence intervals (95% CI).

The studies were of high heterogeneity (*I*^2^ = 96%). Consequently, the random-effects model was used. Our results showed that serum Hcy level in OSA group was 2.40 *μ*mol/l higher than that in control group (95% CI: 0.60 to 4.20, *P* < 0.01) (Figure 2).

### 3.4. Subgroup Analysis

#### 3.4.1. Subgroup Analysis: AHI >= 30

**Figure 3:**
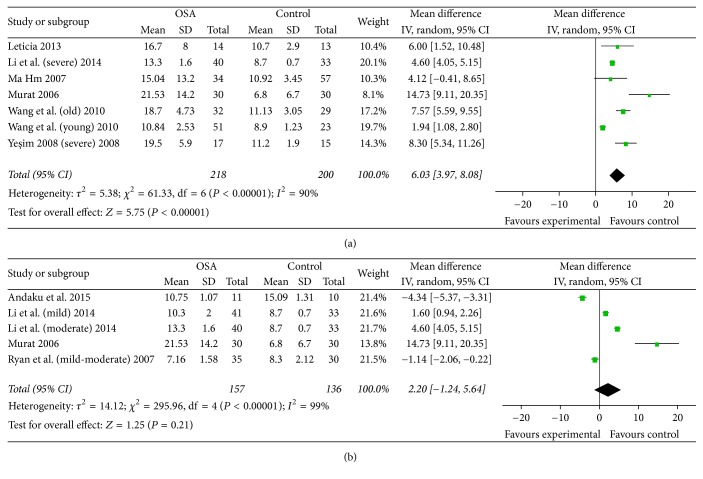
(a) Subgroup analysis and forest plot based on average AHI >= 30. (b) Subgroup analysis and forest plot based on average AHI < 30. Results are expressed as weighted mean difference (WMD) and 95% confidence intervals (95% CI).

Pooled WMD in subgroup with an average AHI >= 30 was more significant, with a value of 6.03 (95% CI: 3.97 to 8.08, *P* < 0.01). In the meantime, the total WMD of subgroup with an average AHI < 30 was not significant, with a value of 2.20 (99% CI: −1.24 to 5.64). There was no significant heterogeneity (*P* = 0.21) (Figure 3).

#### 3.4.2. Subgroup Analysis: BMI >= 30

**Figure 4:**
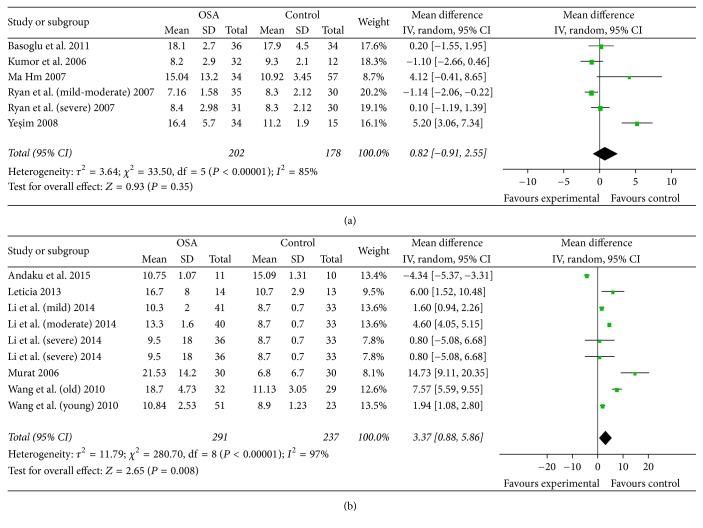
(a) Subgroup analysis and forest plot based on average BMI >= 30. (b) Subgroup analysis and forest plot based on average BMI < 30. Results are expressed as weighted mean difference (WMD) and 95% confidence intervals (95% CI).

Pooled WMD in subgroup with a BMI >= 30 was 0.82 (95% CI: −0.91 to 2.55, *P* = 0.35). There was no significant heterogeneity among two groups. Pooled WMD in subgroup with an BMI < 30 was 3.37 (95% CI: 0.88 to 5.86, *P* = 0.008) (Figure 4).

### 3.5. Sensitivity Analysis

During the sensitivity analysis, results remained steady when each study was withdrawn separately. Remarkably elevated serum Hcy levels were revealed in random-effects model (WMD: 2.4, 95% CI: 0.6 to 4.2, *P* < 0.01). Similar result was revealed in the fixed-effects model (WMD: 1.79, 95% CI: 1.49 to 2.09, *P* < 0.01).

### 3.6. Publication Bias

The present meta-analysis is of slight bias; however, the Begg's (*P* = 0.584) and Egger tests (*P* = 0.947) did not give sufficient evidence of publication bias in this study.

### 3.7. Metaregression Analysis

The result variable was the WMD of serum Hcy levels, and average age, BMI, and publication year were the covariates; they might affect the result in univariate metaregression analysis. Levels of serum Hcy were not significantly linked with following factors, including patients' age (*P* = 0.714), BMI (*P* = 0.418), mean age of control group (*P* = 0.372), control group's BMI (*P* = 0.560), and year of publication (*P* = 0.999).

## 4. Discussion

The association between serum Hcy and OSA has drawn wide attention; however, serum Hcy level in OSA patients compared with health subjects is controversial yet. Our meta-analysis indicated that increased serum Hcy levels were observed in OSA patients without any complication compared with control people (WMD: 2.40, 95% CI: 0.60 to 4.20, *P* < 0.01), particularly in those patients with severe degree OSA (WMD: 6.03, 95% CI: 3.97 to 8.08, *P* < 0.01).

OSA may cause intermittent hypoxia or frequent episodes of arousal. The occurrence of inflammatory reaction in OSA may be associated with increased reactive oxygen species (ROS). In addition, the local inflammatory response caused by recurrent upper airway collapse can also lead to the occurrence of systemic inflammatory response [21]. Homocysteine has a highly reactive sulfhydryl group. The sulfhydryl group easily self-oxidizes [22]. The preceding study demonstrated that more than 98% of homocysteine is in oxidized state [23]. Activated ROS and white blood cells can directly damage the vascular endothelium. With the increase of oxidative stress products in OSA patients, the ability of oxidative stress in vivo decreased [24]. The relationship between atherosclerosis and oxidative stress is also close-knit. Additionally, antioxidants have also been certified to decrease homocysteine-induced endothelial dysfunction, and homocysteine has a deleterious effect on vascular endothelial function mainly through oxidative stress [25]; moreover, OSA and elevated levels of oxidative have significant association. Hence, homocysteine plays an essential role in the process of oxidative stress in patients with OSA. The result of our meta-analysis suggested that serum Hcy levels of OSA patients were higher than that of control people (WMD: 2.40, 95% CI: 0.60 to 4.20, *P* < 0.01), particularly in those patients with severe degree OSA (WMD: 6.03, 95% CI: 3.97 to 8.08, *P* < 0.01). Elevated Hcy levels may be an important cause of severe complications in patients with OSA, particularly in those patients with severe degree OSA.

The previous studies also demonstrated the level of Hcy might have association with OSA [9–18], but some of them surveyed the plasma level of Hcy and others surveyed the serum level of Hcy. Even several meta-analyses did not distinguish plasma Hcy from serum Hcy. In addition, some meta-analysis did not exclude confounded factor, for instance, stroke, coronary heart disease, and metabolic syndrome. These factors might give rise to a degree of experiment data bias. Hence, all participants in our meta-analysis do not have any complication. Our results demonstrated that serum Hcy level was remarkably increased in OSA patients without any complications. Besides, during the sensitivity analysis, results remain steady when each study was withdrawn separately. Above all, our meta-analysis can be deemed as high credibility.

Moreover, we also stratified the data into two subgroups according to BMI (>=30 and <30) and AHI (>=30 and <30), and then subgroup data were analyzed separately. The results suggested that these parameters had remarkable effect on serum Hcy levels in subgroup analysis (AHI >= 30), whereas the result of our meta-analysis showed that BMI had no relationship with the level of serum Hcy in OSA patients (WMD: 0.82, 95% CI: −0.91 to 2.55, *P* = 0.35), and more significant difference was detected in severe OSA patients (WMD: 6.03, 95% CI: 3.97 to 8.08, *P* < 0.01). No significant difference was found in between the OSA patients with an AHI < 30 and controls. Ryan revealed that Pearson's correlation analysis only identified a significant relationship between Hcy levels and HDL-cholesterol (*r* = −0.212, *P* = 0.028). There was no correlation between Hcy and BMI (*P* = 0.642). The conclusion is consistent with the result of our meta-analysis.

Notwithstanding our meta-analysis included relatively high-quality studies, there were still several limitations. Above all, the number of included studies and their sample size were relatively small. More studies with larger sample size would be needed. Secondly, clinical diversity of included studies may bring about statistical heterogeneity and might affect our result. A high heterogeneity among the included studies was present; thus the random-effects models were applied. However, we did not detect the accurate source of heterogeneity. Thirdly, our meta-analysis enrolled 8 case-control trials and 2 cross-sectional trails; it may result in a degree of experimental bias. At last, the detection methods of serum Hcy level vary among included studies, and they may influence the accuracy of serum Hcy level.

Our results suggested that serum Hcy levels in OSA group were higher than in controls. Whether the level of serum Hcy in patients with OSA could become a predictive criterion for the severity of OSA needs to be confirmed by further research.

## 5. Conclusion

Serum Hcy levels and OSA have close-knit and significant association. Analyses demonstrated that patients with OSA had a higher serum Hcy level than healthy controls. In addition, this difference is more significant in moderate or severe OSA patients.

## Figures and Tables

**Table 1:** Characteristics of included studies.

Author	Year	Country	*N* (OG/CG)	NOS
Murat	2006	Turkey	30/30	8
Ma Hm	2007	China	34/57	7
Yeşim	2008	Turkey	34/15	8
Wang et al. (young)	2010	China	51/23	8
Wang et al. (old)	2010	China	32/29	8
Leticia	2013	Brazil	14/13	8
Li et al. (mild)	2014	China	41/33	7
Li et al. (moderate)	2014	China	40/33	7
Li et al. (severe)	2014	China	36/33	7
Kumor et al.	2006	Poland	32/12	8
Ryan et al. (mild-moderate)	2007	Ireland	35/30	8
Ryan et al. (severe)	2007	Ireland	31/30	8
Basoglu et al.	2011	Turkey	36/34	8
Andaku et al.	2015	Brazil	11/10	8

OG: OSA group; CG: control group; NOS: Newcastle-Ottawa Scale; *N*: sample size.

**Table 2:** Participant's characteristics of included study.

Author	HCY mean (SD) (*μ*M)		Mean age (Y)		Mean BMI	
	OG	CG	OG	CG	OG	CG
Murat	21.53 (14.2)	6.8 (6.7)	41.14	42.6	29.63	20.2
Yeşim	16.4 (5.7)	11.2 (1.9)	48.7	43.5	30.8	27.4
Wang et al. (young)	10.84 (2.56)	8.9 (1.23)	42.7	44.7	28.36	25.3
Wang et al. (old)	18.7 (4.73)	11.13 (3.05)	65.8	69.4	23.34	26.85
Leticia	16.7 (8)	10.7 (2.9)	37.2	36	28.8	26.9
Li et al. (mild)	10.3 (2)	8.7 (0.7)	42.6	44.7	24.4	25.1
Li et al. (moderate)	13.3 (2.6)	8.7 (0.7)	40.5	44.7	27.3	25.1
Li et al. (severe)	9.5 (1.8)	8.7 (0.7)	41.8	46.67	26.95	25.84
Ma Hm	15.04 (13.2)	10.92 (3.46)	46.27	49	35	31
Kumor et al.	8.2 (2.9)	9.3 (2.1)	51.3	42.8	30.6	26.9
Ryan et al. (mild-moderate)	7.16 (1.58)	8.3 (2.12)	42	41	32.9	30.7
Ryan et al. (severe)	8.4 (2.98)	8.3 (2.12)	43	41	32.1	30.7
Basoglu et al.	18.1 (2.7)	17.9 (4.5)	50	49.7	33.5	34.5
Andaku et al.	10.75 (1.07)	15.09 (1.31)	42.36	43	26.65	24.14

OG, OSA group; CG, control group; Hcy: homocysteine; SD, standard deviation; BMI, body mass index.
